# First-principles calculations of quartz–coesite interfaces

**DOI:** 10.1107/S1600576725000093

**Published:** 2025-02-01

**Authors:** Tim Schaffrinna, Victor Milman, Björn Winkler

**Affiliations:** a Institute of Geosciences Goethe University Frankfurt Germany; b Dassault Systèmes BIOVIA, Cambridge, United Kingdom; HPSTAR and Harbin Institute of Technology, People’s Republic of China

**Keywords:** martensitic transformations, solid–solid interfaces, high-resolution transmission electron microscopy, HRTEM, quartz–coesite interfaces, strain-induced transitions

## Abstract

Atomistic interface structures compatible with periodic boundary conditions for the strain-induced subsolidus martensitic transition between quartz and coesite have been investigated. We identified layers of atoms that remained unchanged in terms of neighbor interactions throughout the transformation. Our analysis revealed that the orientation relationships between quartz and coesite, namely (1011)_Qz_||(010)_Coe_ and (1321)_Qz_||(010)_Coe_, are consistent with experimental observations. Using density-functional-theory-based tight-binding model cal­culations, we determined an interface energy of approximately 660 mJ m^−2^ for these interfaces and strain energies of 196 (6) and 2760 (160) J mol^−1^ atom^−1^ for the (1321)_Qz_||(010)_Coe_ and (1011)_Qz_||(010)_Coe_ oriented interfaces, respectively. To visualize these interface structures and facilitate their identification in experiments, we simulated high-resolution transmission electron microscopy images and electron diffraction patterns.

## Abstract 2

Atomistic models compatible with periodic boundary conditions have been employed to understand quartz–coesite interfaces.

## 1. Introduction

Coesite is a high-pressure polymorph of SiO_2_ and is an important indicator of phenomena such as ultra-high pressure metamorphism and hypervelocity impacts. Under equilibrium conditions, the transition from quartz to coesite is known to be a first-order reconstructive phase transformation (Dmitriev *et al.*, 1998 ▸). However, under extreme conditions such as shock compression, studies have indicated that coesite can form directly from shocked quartz in subsolidus conditions (Campanale *et al.*, 2019 ▸, 2021 ▸; Richter *et al.*, 2016 ▸).

Recently, a mechanism for a martensitic transformation path from quartz to coesite under strain has been shown to exist (Schaffrinna *et al.*, 2024 ▸). Martensitic phase transitions are solid-state phase transformations that involve a rapid change in the crystal structure at certain temperatures or under stress conditions without a change in chemical composition. Martensitic transformations involve small atomic displacements and the product phase often has a well defined lattice orientation relationship with respect to the starting phase. Experiments have revealed possible {1011}_Qz_||(010)_Coe_ and {1321}_Qz_||(010)_Coe_ orientation relationships between quartz and coesite in shocked samples (Campanale *et al.*, 2019 ▸, 2021 ▸). In addition, there is an invariant plane in martensitic transformations, *i.e.* a plane in which atoms are not significantly displaced (Therrien *et al.*, 2020 ▸; Therrien & Stevanović, 2020 ▸).

The changes in the contributions to the free energy during the transition from quartz to coesite, such as the interface energy and the elastic strain energy (caused by the lattice mismatch between quartz and coesite), are difficult to measure experimentally. However, morphological quantities such as lattice parameters, orientation relationships or planar defect features (PDFs) can be obtained experimentally from high-resolution transmission electron microscopy (HRTEM) or diffraction experiments. In this study, we investigate the interfaces in quartz which has been martensitically transformed into coesite with the (1011)_Qz_||(010)_Coe_ and (1321)_Qz_||(010)_Coe_ orientation relationships between quartz and coesite determined by atomistic model calculations. We provide interface and strain energies derived from density functional theory tight-binding (DFTB) and empirical force-field model calculations, and show that the interface structures are stable against small distortions. We present simulated HRTEM interface images and electron diffraction patterns in order to facilitate their detection in future experimental studies.

## 2. Methods

Our approach is based on a combination of geometric minimization of the transformation pathway and subsequent construction of the interface structures in conjunction with atomistic modeling. For the latter, established methods based on density functional theory (DFT) (Hohenberg & Kohn, 1964 ▸; Perdew *et al.*, 1996 ▸; Clark *et al.*, 2005 ▸), DFTB (Hourahine *et al.*, 2020 ▸) and empirical force-field calculations (Gale & Rohl, 2003 ▸) are employed. We first describe how the interface cells are constructed from the transition pathway and then we show how the correct energies are obtained.

The process of the martensitic transformation from quartz to coesite involves a series of structural changes that were simulated using the *p2ptrans* software package (Therrien *et al.*, 2020 ▸; Schaffrinna *et al.*, 2024 ▸). This package utilizes an approach in which individual atoms are matched between an initial and a final crystal structure. By defining a transformation cell (*i.e.* the smallest unit cell necessary to describe the transition using periodic boundary conditions) and generating intermediate structures, the software allows the computation of details of the transformation process, including Bain strain, space groups and coordinates of uniformly strained planes. To enhance the efficiency of the package, we improved it (Schaffrinna *et al.*, 2024 ▸) by replacing the Hungarian algorithm with the more efficient Jonker–Volgenant algorithm (Crouse, 2016 ▸; Jonker & Volgenant, 1987 ▸). This modification significantly improves the speed of the atom-to-atom assignment process between initial and final structures (Therrien, 2023 ▸).

The search for a transformation cell initially involves a significant expansion of the unit cells of the models for both the initial and the final structures.

In our previous study (Schaffrinna *et al.*, 2024 ▸), we determined that 4320 atoms are needed for a successful mapping of the quartz structure onto the coesite structure, comprising 480 unit cells of quartz and 180 primitive unit cells of coesite. Once the mapping process was completed, we were able to identify the smallest unit cell compatible with periodic boundary conditions necessary to describe the transition sequence. This minimal cell is oblique and contains 24 SiO_2_ formula units and will be referred to as the ‘transformation cell’.

**Figure 1:**
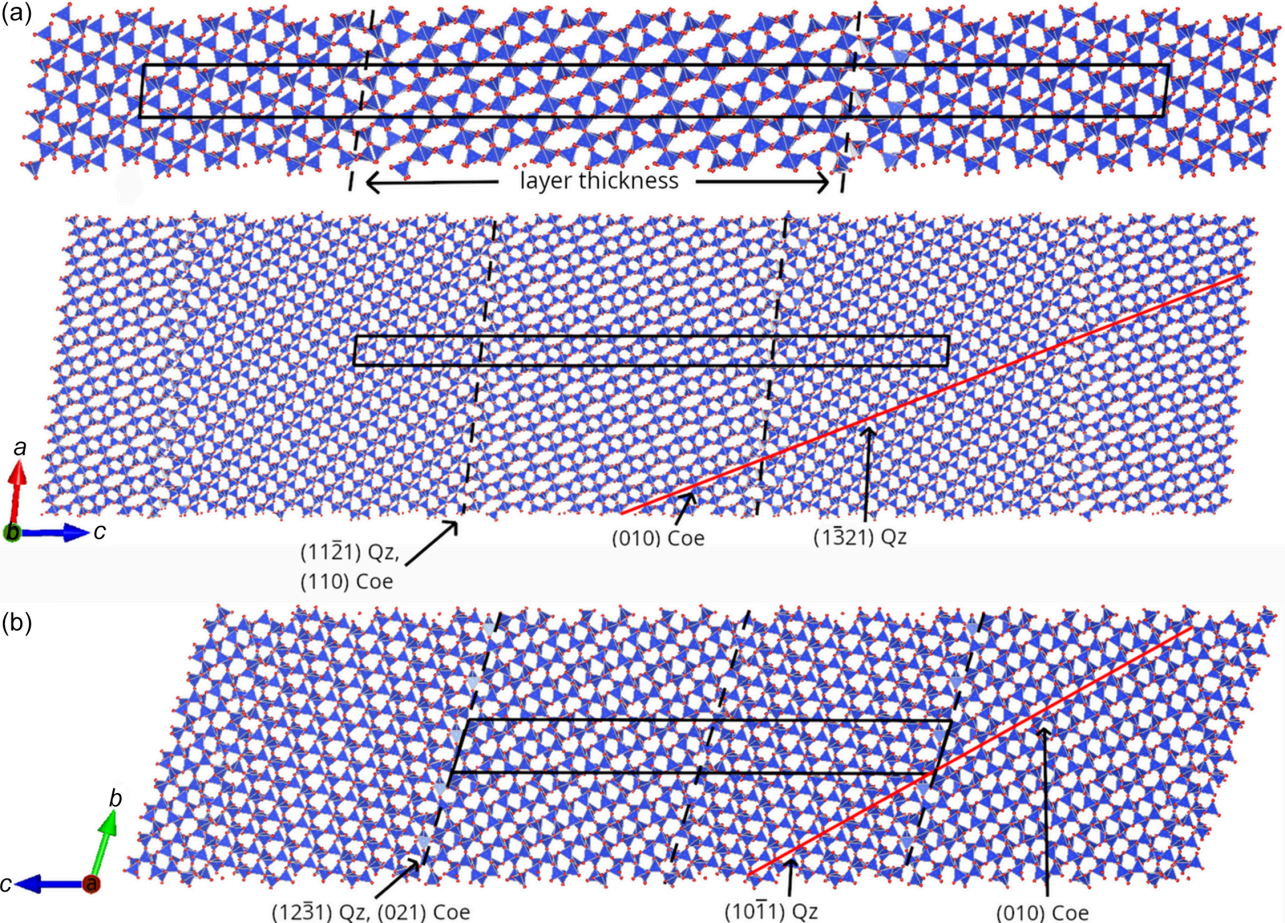
Interface quartz–coesite structures with periodic boundary conditions. Red lines are lattice planes with a common orientation. Interfaces are shown with dashed lines. (*a*) Details of the magnified interface cell (upper) and the quartz–coesite orientation relationship (lower). Coesite (middle layer) oriented (1321)_Qz_||(010)_Coe_ in quartz (left and right layers of the interface cell, shown as black lines). The interface plane has the orientation (1121)_Qz_||(110)_Coe_. (*b*) Coesite (left layer in the interface cell) oriented (1011)_Qz_||(010)_Coe_ in quartz. The interface plane has the orientation (1231)_Qz_||(021)_Coe_. The atom positions correspond to the equilibrium positions. Oxygen atoms are shown as red spheres, while the positions of silicon atoms are inside blue tetrahedrons.

A martensitic subsolidus phase transition between an initial crystal structure, quartz, and a final crystal structure, coesite, implies that there is a set of atoms within a plane in the transformation cell that does not exchange neighboring atoms during the transformation. Such planes are termed ‘invariant planes’. On the basis of our previous calculations (Schaffrinna *et al.*, 2024 ▸), we studied interface models, which have an orientation relationship where (1011)_Qz_||(010)_Coe_ or where (1321)_Qz_||(010)_Coe_, consistent with experimental observations (Fig. 1 ▸; Campanale *et al.*, 2021 ▸). In order to study the orientation relations, we created sandwich models with varying spacings between the invariant planes, at which the coesite structure directly transitions into the quartz structure. This approach results in the creation of two interfaces, which separate slabs of quartz and coesite.

This has been done for various layer thicknesses between the interfaces (Fig. 1 ▸). The supercells employed in calculations with periodic boundary conditions and containing the interface structures will be called ‘interface cells’ hereafter.

We carried out full geometry optimizations of the interface cells with *N* atoms having different layer thicknesses of the quartz and coesite components to calculate the energy of formation, *E*_f_. The geometry optimizations yielded *E*_tot_, the total energy of the fully relaxed interface cell. *E*_Qz_ and *E*_Coe_ in equation (1) are the total energies per atom of the fully relaxed quartz and coesite structures, respectively. *x* in equation (1) represents the phase fraction of the quartz structure in the interface cell, and then *E*_f_ is defined as (Wang *et al.*, 2007 ▸)
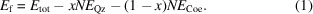
The formation energy of the interface cells [equation (1)] contains contributions from both the interface energy and the elastic strain energy due to the lattice mismatch between quartz and coesite.

Specifically, the energy of formation [equation (1)] can be expressed as (Wang *et al.*, 2007 ▸)
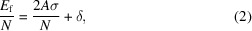
where *A* represents the area of the interface, σ is the interface energy per unit area and δ is the strain energy per atom (the factor of two is due to the fact that there are two interfaces per interface cell).

The asymptotic behavior of the energy of formation shows that, as the distance from the interface increases, the contributions of the interface energy (which represents the energy cost of maintaining the interface) decrease, while the strain energy (which reflects the energy associated with the deformation of the material due to the interface) approaches a limit. As the distance between the interfaces increases, the interface cell tends to become more like a bulk material, where the strain energy per atom becomes the dominant factor at large distances, leading to a stabilization of the formation energy as the influence of the interface decreases.

In order to separate the interface energy from the strain energy we carried out geometry optimizations for a range of interface cell sizes and subsequently fitted the computed *E*_f_ with equation (2). Though this method is conceptually straightforward, it can be computationally intensive in state-of-the-art DFT calculations (Hohenberg & Kohn, 1964 ▸; Wang *et al.*, 2007 ▸). Therefore, we employed computationally less demanding model calculations, sacrificing accuracy but allowing much larger system sizes.

Full geometry optimizations of the interface cells were performed with DFT-based tight-binding calculations. This is a semiempirical method that is 2–3 orders of magnitude faster than the conventional plane wave/pseudopotential-based DFT calculations, but has an accuracy similar to DFT when a proper Slater–Koster parametrization of the pairwise element–element interactions is used. We have performed these calculations using the *DFTB*+ program package (Hourahine *et al.*, 2020 ▸) using the pbc Slater–Koster dataset (Köhler *et al.*, 2001 ▸; Sieck *et al.*, 2003 ▸). The repulsive potentials for this set were re-parameterized (Panosetti *et al.*, 2020 ▸) to match the equations of state and elastic coefficients of quartz, coesite and stishovite with experimental (Ross *et al.*, 1990 ▸; Yamanaka *et al.*, 2002 ▸; Levien *et al.*, 1980 ▸; Glinnemann *et al.*, 1992 ▸; Hazen *et al.*, 1989 ▸) and theoretical DFT (Dong *et al.*, 2015 ▸; Winkler & Milman, 2014 ▸) data. The accuracy of this parametrization is well established (Schaffrinna *et al.*, 2024 ▸).

Additional full geometry optimizations were performed with *GULP* (Gale & Rohl, 2003 ▸) where an empirical three-body force field with a core–shell repulsion for the oxygen atoms was employed (Table 1 ▸). The accuracy of results obtained with this force field is also well established (Sanders *et al.*, 1984 ▸).

To benchmark the *DFTB*+ and force-field models, we carried out first-principles calculations within the framework of DFT, employing the Perdew–Burke–Ernzerhof (PBE) exchange-correlation functional and the plane wave/pseudopotential approach implemented in the *CASTEP* simulation package (Hohenberg & Kohn, 1964 ▸; Perdew *et al.*, 1996 ▸; Clark *et al.*, 2005 ▸). On-the-fly ultrasoft pseudopotentials generated using the descriptors in the 2017 revision 2 *CASTEP* database were employed in conjunction with plane waves up to a kinetic energy cutoff of 630 eV. The accuracy of these pseudopotentials is well established (Lejaeghere *et al.*, 2016 ▸). A Monkhorst–Pack grid was used for Brillouin zone integrations (Monkhorst & Pack, 1976 ▸). We used a distance between grid points of <0.023 Å^−1^. The convergence criteria for geometry optimization included an energy change of <5 × 10^−6^ eV atom^−1^ between steps, a maximal force of <0.01 eV Å^−1^ and a maximal component of the stress tensor <0.02 GPa. The accuracy of these settings for the calculation of structures and properties of SiO_2_ phases is illustrated in the literature (Bosak *et al.*, 2009 ▸; Winkler & Milman, 2014 ▸; Lobanov *et al.*, 2022 ▸).

Elastic coefficients and their statistical errors were obtained from linear fitting of the stress–strain dependencies (Milman & Warren, 2001*a* ▸; Milman & Warren, 2001*b* ▸) for *DFTB*+ calculations and by an analytic derivation in the *GULP* calculations. Elastic tensor analysis was performed with the *ELATE* program package (Gaillac *et al.*, 2016 ▸).

Simulated HRTEM images and electron diffraction images were computed with the *ReciPro* package (Seto & Ohtsuka, 2022 ▸). We used a resolution of 8 pm pixel^−1^ and an image size of 2048 × 2048 pixels. Images were simulated with 200 eV acceleration voltage with an FWHM of 0.50 eV, a sample thickness of 50 nm, a defocussed electron beam of 50 nm, 700 simulated waves and an open aperture.

## 3. Results

An initial set of calculations confirmed the overall accuracy of the DFT–GGA–PBE, DFT-based tight-binding and *GULP* force-field calculations (Table 2 ▸), consistent with earlier studies (Bosak *et al.*, 2009 ▸; Winkler & Milman, 2014 ▸; Lobanov *et al.*, 2022 ▸; Schaffrinna *et al.*, 2024 ▸). It is well known that DFT–GGA–PBE underestimates the electron–electron exchange correlations and therefore overestimates the lattice parameters. The same applies for DFTB calculations, while fully empirical force fields, like the one used here, may underestimate the lattice parameters. These minor deviations from the experimentally determined lattice parameters do not affect the conclusions of this study.

### 3.1. Interface structure

The interface plane relationship for quartz and coesite is (1121)_Qz_||(110)_Coe_ where (1321)_Qz_||(010)_Coe_, and (1231)_Qz_||(021)_Coe_ is the interface plane relationship for (1011)_Qz_||(010)_Coe_ (dashed lines in Fig. 1 ▸). These are shown as lines in Fig. 1 ▸.

The first major result is the explicit confirmation of an invariant layer of atoms in the interface cells, in which the nearest neighbors are maintained throughout the diffusionless transition from quartz to coesite. The lattice parameters for the geometry optimized interface cells are given in Table S1 of the supporting information.

A typical interface sequence is shown in Fig. 1 ▸. We observe an ordered, relaxed structure at the interface after geometry optimizations, consistent with experiments (Campanale *et al.*, 2021 ▸). Specifically, a geometry optimized interface structure obtained with *CASTEP* had no force component on any atom >0.012 eV Å^−1^.

### 3.2. Interface energy

**Figure 2:**
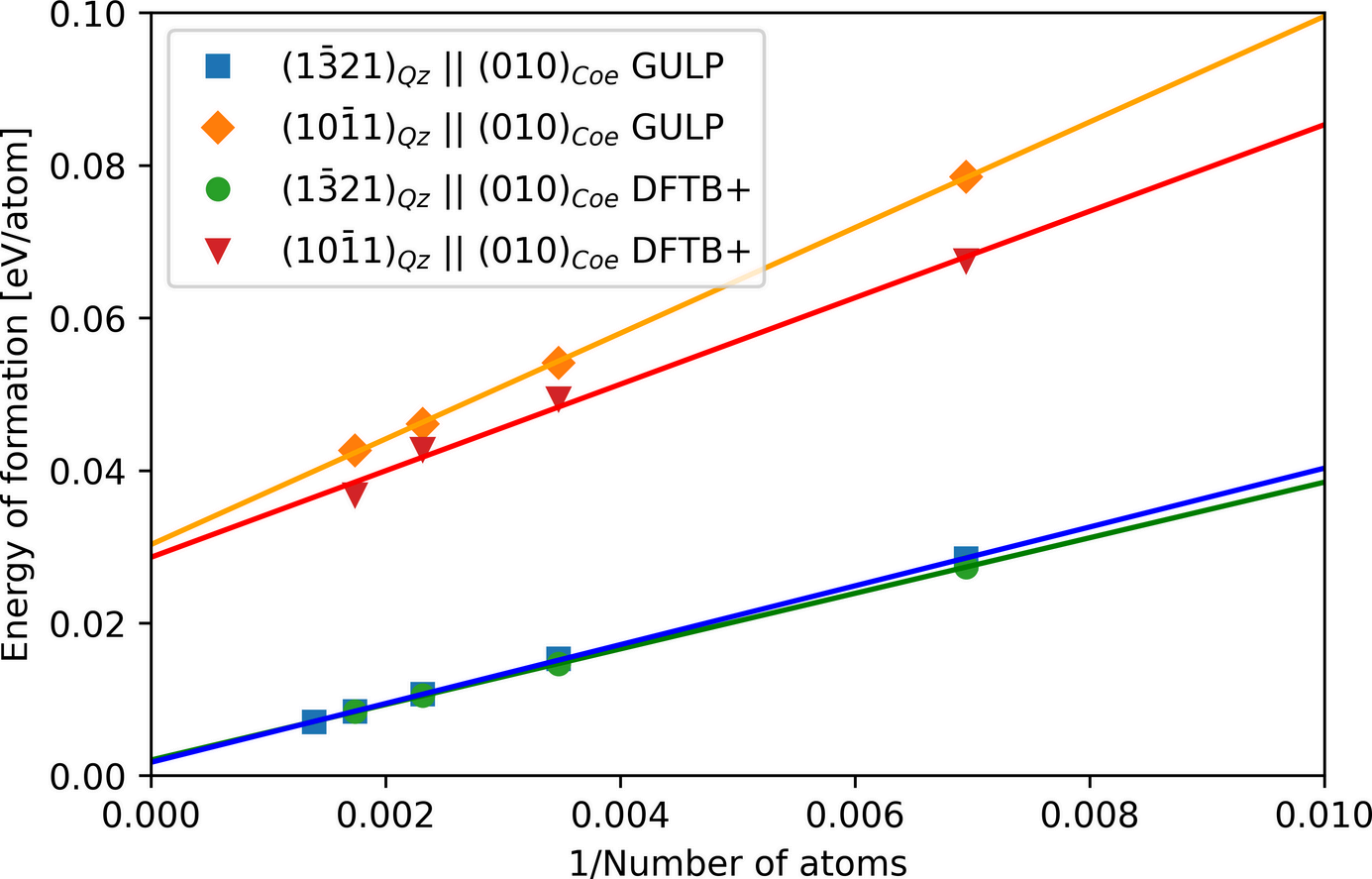
Energy of formation of fully relaxed interface cells as a function of the interface cell size fitted to equation (2). All values are normalized to an average of the ground-state energy of quartz and coesite at ambient pressure. Strain energies δ are given by the energies at *x* = 0.

In atomistic model calculations with periodic boundary conditions, a spurious interaction between closely spaced interfaces can bias both interface and strain energies, as well as structural characteristics. When the spacing between interfaces is sufficiently large, the calculated interface energy should be independent of the size of the interface cell. To verify this, we conducted comprehensive geometry optimizations with varying interface spacings, as shown in Fig. 2 ▸.

With our *DFTB*+ model calculations, we obtained similar interface energies of about 660 mJ m^−2^ for the two different interface structures (Table 3 ▸). Although the interface energies are almost identical for both interface structures, their strain energies differ by an order of magnitude. The values obtained with the *DFTB*+ model are in good agreement with the values based on the force-field calculations (Fig. 2 ▸), and the values computed here are similar to other interface systems (Table 3 ▸).

A second major result is that the interface cells for both orientations are stable with respect to small distortions. This is evident from the elastic stiffness tensors (Tables S2 and S3) as they only have positive eigenvalues and are converged with respect to the interface cell size, if two or more transformation cells of quartz and coesite are combined. A validation of the accuracy of the elastic stiffness coefficients obtained by the *DFTB*+ calculations against a DFT–GGA–WC model for various SiO_2_ structures, even with unusual structural features, is given by Schaffrinna *et al.* (2024 ▸). Here we further confirm the internal consistency by comparison to a well established empirical force-field model (Sanders *et al.*, 1984 ▸). *DFTB*+ and the force-field model yield similar elastic stiffness tensors (Fig. S1). These results imply that the interface structures are mechanically stable and can be preserved.

### 3.3. HRTEM simulations

**Figure 3:**
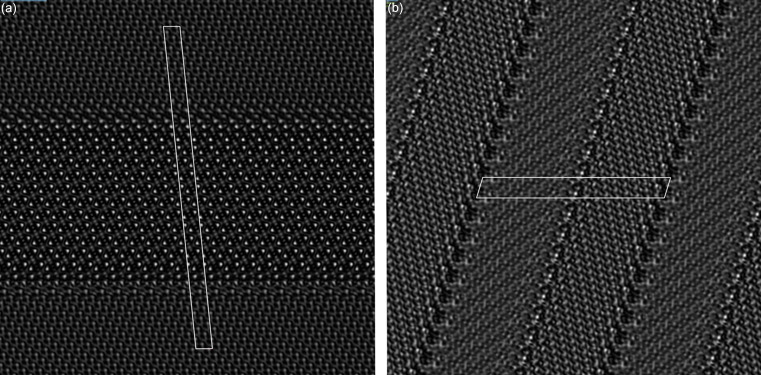
Simulated HRTEM images of the interface structures in (*a*) Fig. 1 ▸(*a*) and (*b*) Fig. 1 ▸(*b*). Image width is 16.4 nm in both cases. The interface cells are highlighted as white boxes.

HRTEM can be used to visualize the boundary between phases as well as interface structures. This is important because the atomic arrangement at the interface can significantly influence the physical and chemical properties of the material. To allow a rapid identification of quartz–coesite interfaces in samples at the nanoscale, we have generated HRTEM images of the interface structures (Fig. 3 ▸). The simulated HRTEM images show the layered structure with the interfaces at the nanoscale, which is comparable to observed layers of quartz and coesite in TEM images of shocked samples (Campanale *et al.*, 2019 ▸).

**Figure 4:**
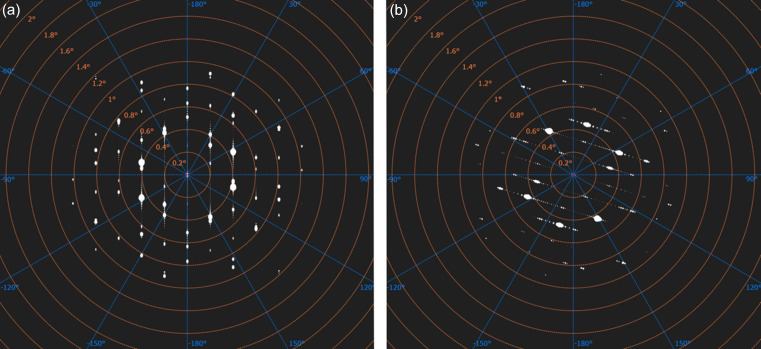
Simulated electron diffraction patterns of the interface structures in (*a*) Fig. 1 ▸(*a*) and (*b*) Fig. 1 ▸(*b*).

In addition to the HRTEM images, we also simulated electron diffraction patterns of the interface structure to facilitate the determination of the crystallographic orientation of quartz–coesite interfaces (Fig. 4 ▸). The electron diffraction patterns of the interface cells show slightly distorted peaks in one direction, which has also been experimentally observed in TEM electron diffraction (Campanale *et al.*, 2019 ▸) in shocked samples.

## 4. Discussion

It is widely accepted that varying levels of stress can greatly impact the thresholds for metamorphic reactions (Wheeler, 2014 ▸). Through a series of experiments (Richter *et al.*, 2016 ▸; Zhou *et al.*, 2005 ▸; Ren, 2022 ▸) it has been demonstrated that differential stress can trigger a phase transition from quartz to coesite even at pressures lower than the equilibrium pressure. We have developed an atomistic model to quantitatively understand the subsolidus transformation induced by stress (Schaffrinna *et al.*, 2024 ▸), allowing us to quantitatively analyze the evolving microstructure due to this process. The interface structures could be built by identifying layers of atoms that do not exchange neighbors during the transition. These interface layers are not involved in the slipping process, which occurs parallel to the planes near {1011} and {1321} that remain invariant during the quartz–coesite transformation (Schaffrinna *et al.*, 2024 ▸). The invariant planes along the slipping process are commonly observed as PDFs in shocked quartz samples. However, there are currently no studies providing HRTEM images of the interfaces with an atomic resolution.

Our atomistic model explains the microstructure in shocked quartz due to a subsolidus martensitic transformation. The predicted microstructure closely matches the observed structural characteristics in shocked natural samples and uniaxial compression experiments. Specifically, we have created interface structures where sets of planes in quartz near {1011} and {1321} are parallel to the (010) plane in coesite, which were experimentally observed in natural shocked samples (Campanale *et al.*, 2021 ▸). In all transition models we have calculated, there are only these two orientation relationships. The orientation relationships in our model provide predictions for future EBSD analyses of quartz–coesite interfaces.

The calculated strain energies and interface energies based on *DFTB*+ and force-field model calculations are within the ranges of other interface systems (Table 3 ▸) and show that the data in this study are similar to other DFT-based studies. The interface energies of about 660 mJ m^−2^ determined using *DFTB*+ model calculations for both orientation relations imply that the orientation after the transformations is not determined by the interface energies. In contrast, the strain energy for the (1011)_Qz_||(010)_Coe_ interface structures is an order of magnitude higher than that for the (1321)_Qz_||(010)_Coe_ interface structures. While the *c* lattice parameters of the quartz and coesite transformation cells only decrease by approximately −8% for the (1321)_Qz_||(010)_Coe_ interface, the *b* and *c* lattice parameters change by approximately −13% and 11% for the (1011)_Qz_||(010)_Coe_ interface, thus resulting in a higher strain in the interface cells (Table S1).

This suggests that in experiments the {1321}_Qz_||(010)_Coe_ orientation should dominate. All interface structures are stable against small distortions, as indicated by positive eigenvalues of all elastic stiffness coefficient tensors, suggesting that these interfaces are a possible explanation at the atomic level for the orientation relationships found in naturally shocked quartz samples (Campanale *et al.*, 2019 ▸, 2021 ▸).

The presence of these invariant plane families in both shock experiments and naturally shocked quartz strongly suggests that the transformation mechanism (Schaffrinna *et al.*, 2024 ▸) and the corresponding microstructure, identified in this study, is the process occurring in nature.

## Supplementary Material

Lattice parameters and elastic stiffness coefficients of interface cells. DOI: 10.1107/S1600576725000093/iu5073sup1.pdf

## Figures and Tables

**Table:** The maximum range for the interatomic potentials is 100000 Å.

Element	Type	Atomic mass (a.u.)	Charge (e)	Covalent radius, ionic radius, VDW radius (Å)
O	Core	16.00	0.86902	0.73, 0.0, 1.36
O	Shell	0.00	−2.86902	0.73, 0.0, 1.36
Si	Core	28.09	4.00000	1.20, 0.0, 2.1

**Table:** 

Elements	Potential	Parameter	Value	Cutoffs (min, max) (Å)
Si_core_–O_shell_	Buckingham	*A*	1283.9070 eV	(0.0, 10.0)
		ρ	0.32052 Å	
		*C*	10.661580 eV Å^6^	
O_shell_–O_shell_	Buckingham	*A*	22764.000 eV	(0.0, 12.0)
		ρ	0.149 Å	
		*C*	27.879 eV Å^6^	
O_core_–O_shell_	Spring	*k*2	74.92 eV Å^−2^	(0.0, 0.8)
		*k*4	74.92 eV Å^−4^	

Si_core_–O_shell_–O_shell_	Harmonic	Three-body cnst	2.09724 eV rad^−2^	(0.0, 1.9)
		Three-body angle	109.47°	(0.0, 1.9)

**Table 2:** Comparison of experimentally determined lattice parameters with those computed with DFT, DFT tight binding and empirical force fields of quartz and coesite at ambient pressure

	Quartz				Coesite			
	Experiment†	DFT–GGA–PBE (*CASTEP*)	DFTB (*DFTB*+)	Empirical force field (*GULP*)	Experiment‡	DFT–GGA–PBE (*CASTEP*)	DFTB (*DFTB*+)	Empirical force field (*GULP*)
*a* (Å)	4.916	5.038	5.002	4.834	7.136	7.270	7.285	7.025
*b* (Å)	4.916	5.038	5.002	4.834	12.369	12.540	12.689	12.290
*c* (Å)	5.409	5.525	5.470	5.344	7.174	7.255	7.335	7.115
β (°)	120.00	120.00	120.00	120.00	120.34	120.07	120.02	122.48
*V* (Å^3^)	113.199	121.429	118.528	108.162	546.439	572.347	587.046	518.235
ρ (g cm^−3^)	2.644	2.465	2.525	2.767	2.921	2.789	2.719	3.080

† Gualtieri (2000 ▸).

‡ Levien & Prewitt (1981 ▸).

**Table 3:** Interface energies and strain energies of the interface structures calculated with *DFTB*+ and *GULP* at ambient pressure compared with other interface systems

Interface	Method	Interface energy (mJ m^−2^)	Strain energy (J mol^−1^ atom^−1^)	*R* ^2^
{1321}_Qz_||(010)_Coe_	*DFTB*+	652 (2)	196 (6)	0.99997
{1321}_Qz_||(010)_Coe_	*GULP*	634 (4)	168 (9)	0.99988
{1011}_Qz_||(010)_Coe_	*DFTB*+	660 (40)	2760 (160)	0.99009
{1011}_Qz_||(010)_Coe_	*GULP*	866 (10)	2920 (40)	0.99972
β′-Mg_5_Si_6_–α-Al interfaces†	DFT	100–449	390–1610	
Mg–MgO interface‡	DFT	743–1048	2509–3184	
Al_3_*M*–Al interface§	DFT	78–231	125–1042	
γ-Al_2_O_3_–Al interface¶	DFT	1040–3450	75–1070	
SiO_2_–Al interface††	DFT	1345		
Cu–Fe–*X* alloys‡‡	DFT	288–869		
Cu–Fe interfaces§§	DFT	350–530		

† Wang *et al.* (2007 ▸).

‡ Xu *et al.* (2015 ▸).

§ Song *et al.* (2023 ▸).

¶ Zhang *et al.* (2023 ▸).

†† Zhang *et al.* (2021 ▸).

‡‡ Wang *et al.* (2020 ▸).

§§ Garrett & Race (2021 ▸).

## Data Availability

All software written by us for this study and all data produced in the present study are available upon request from TS.
