# Supplementary material for: First-principles calculations of quartz–coesite interfaces
Source: J Appl Crystallogr. 2025 Feb 1;58(Pt 1):180–6. doi: 10.1107/S1600576725000093 (PMC11798512; doi:10.1107/S1600576725000093)
Supplement: Supplementary file 1 [file j-58-00180-sup1.pdf]

JOURNAL OF
APPLIED
CRYSTALLOGRAPHY

Volume 58 (2025)

Supporting information for article:

First-principles calculations of quartz–coesite interfaces

Tim Schaffrinna, Victor Milman and Björn Winkler

Supporting Information: First-principles calculations of quartz-coesite interfaces

Tim Schaffrinna,[a] Victor Milman[b] and Björn Winkler[a]

[a] *Institute of Geosciences, Goethe University, Frankfurt a.M., Germany, and*

[b] *Dassault Systèmes BIOVIA, Cambridge, United Kingdom*

Table S1. *Lattice parameters of DFTB+- and GULP-fully relaxed interface cells at ambient pressure. $x + y$ denote the number of x and y transformation cells of quartz and coesite, respectively.*

	Structure	a (Å)	b (Å)	c (Å)	α (°)	β (°)	γ (°)	V (Å^3)	ρ (g cm^{-3})
					$\{1\bar{3}21\}_{\mathrm{Qz}}\|\|(010)_{\mathrm{Coe}}$				
DFTB+	Quartz	7.412	7.412	18.571	80.68	80.68	71.53	948.343	2.525
	1+1	7.432	7.310	36.495	79.69	81.34	73.42	1857.041	2.578
	2+2	7.438	7.329	71.768	80.99	81.98	73.49	3685.795	2.599
	3+3	7.421	7.335	107.351	81.45	82.13	73.56	5489.525	2.605
	4+4	7.413	7.339	142.947	81.68	82.21	73.60	7345.053	2.608
	Coesite	7.316	7.335	17.089	84.03	84.16	75.58	880.572	2.719
GULP	Quartz	7.209	7.209	18.022	80.21	80.21	71.03	866.122	2.765
	1+1	7.206	7.118	34.394	78.40	81.62	72.34	1639.704	2.921
	2+2	7.171	7.112	68.808	79.42	81.82	72.50	3275.828	2.924
	3+3	7.164	7.118	102.960	79.93	82.03	72.55	4910.877	2.926
	4+4	7.158	7.117	137.372	80.18	82.07	72.57	6550.669	2.924
	Coesite	7.078	7.114	16.213	81.72	84.70	74.51	777.285	3.081
					$\{10\bar{1}1\}_{\mathrm{Qz}}\|\|(010)_{\mathrm{Coe}}$				
DFTB+	Quartz	7.423	10.247	13.235	106.07	97.33	95.76	948.298	2.525
	1+1	7.335	9.417	27.726	105.66	102.50	92.06	1791.332	2.673
	2+2	7.328	9.416	55.599	106.14	102.07	92.40	3583.089	2.673
	3+3	7.315	9.404	83.824	106.36	101.87	92.40	5383.950	2.668
	4+4	7.318	9.408	111.590	106.42	101.68	92.43	7176.240	2.669
	Coesite	7.285	8.977	14.644	107.86	104.18	90.18	880.554	2.719
GULP	Quartz	7.209	9.936	12.794	106.02	97.28	95.20	866.123	2.765
	1+1	7.074	9.036	26.460	106.13	102.06	91.72	1581.922	3.028
	2+2	7.109	9.024	52.854	106.34	101.31	92.51	3172.913	3.019
	3+3	7.120	9.014	79.365	106.40	101.10	92.77	4766.678	3.014
	4+4	7.120	9.008	106.080	106.51	101.17	92.93	6358.267	3.013
	Coesite	7.026	8.595	13.793	106.36	102.12	92.05	777.352	3.081

Table S2. *Elastic coefficients (in GPa) of the* $\{1\bar{3}21\}_{\mathrm{Qz}}||(010)_{\mathrm{Coe}}$ *interface structures at ambient pressure.* $x+y$ *denotes the number of* x *and* y *transformation cells corresponding to the quartz and coesite structure, respectively.*

	DFTB+			GULP			
C_{ij}	1+1	2+2	3+3	1+1	2+2	3+3	4+4
c_{11}	130(2)	138(2)	133(2)	143	135	135	137
c_{12}	31(2)	32(2)	30(1)	49	34	36	34
c_{13}	25(1)	30(2)	28(1)	38	45	42	44
c_{14}	-2(1)	0(1)	1(1)	-2	3	2	3
c_{15}	9(2)	2(2)	2(1)	1	3	0	-1
c_{16}	-11(2)	6(1)	8(1)	-6	-2	1	2
c_{22}	130(1)	146(2)	148(1)	184	172	172	171
c_{23}	31(0)	42(1)	41(1)	34	33	32	32
c_{24}	-1(1)	13(1)	16(1)	15	18	18	18
c_{25}	8(1)	2(2)	1(1)	4	7	6	7
c_{26}	-1(1)	-4(1)	-5(1)	19	21	23	23
c_{33}	109(1)	98(2)	97(1)	109	96	103	101
c_{34}	1(1)	2(1)	3(1)	1	3	2	2
c_{35}	-1(1)	4(1)	5(1)	-4	-9	-4	-5
c_{36}	-3(1)	-4(1)	-4(1)	0	2	0	0
c_{44}	44(2)	43(1)	43(1)	30	31	30	30
c_{45}	0.1(8)	0(1)	0(1)	0	-1	-2	-2
c_{46}	2(1)	0(1)	0(1)	0	1	1	2
c_{55}	44(2)	42(2)	42(1)	42	35	38	38
c_{56}	-4(2)	0(1)	0(1)	3	6	5	5
c_{66}	36(1)	39(1)	40(1)	46	43	44	44
B	59(1)	64(1)	62(2)	73(2)	67(3)	68(2)	67(2)

Table S3. *Elastic coefficients (in GPa) of the* $\{10\bar{1}1\}_{\mathrm{Qz}}||(010)_{\mathrm{Coe}}$ *interface structures at ambient pressure.* $x+y$ *denotes the number of* x *and* y *transformation cells corresponding to the quartz and coesite structure, respectively.*

	DFTB+			GULP			
C_{ij}	1+1	2+2	3+3	1+1	2+2	3+3	4+4
c_{11}	139(5)	131(2)	133(2)	126	130	132	133
c_{12}	50(5)	42(2)	41(2)	54	59	60	61
c_{13}	55(5)	44(2)	43(2)	48	45	44	43
c_{14}	4(3)	-2(2)	-6(2)	-5	-6	-7	-5
c_{15}	2(2)	7(2)	11(2)	0	10	12	14
c_{16}	-9(4)	-9(1)	-8(2)	0	-3	-5	-5
c_{22}	161(5)	147(2)	140(2)	137	127	123	118
c_{23}	70(4)	55(2)	51(2)	55	46	47	46
c_{24}	2(2)	4(1)	4(2)	0	3	4	2
c_{25}	3(2)	4(1)	6(2)	-2	10	12	12
c_{26}	6(4)	3(1)	2(2)	6	-1	-5	-7
c_{33}	151(4)	142(2)	135(3)	118	105	107	108
c_{34}	-3(3)	-6(1)	-6(2)	6	3	3	5
c_{35}	-7(2)	-2(2)	-3(2)	1	3	3	-1
c_{36}	-3(3)	-6(1)	-5 (2)	3	5	4	3
c_{44}	56(2)	54(2)	54(2)	50	50	50	48
c_{45}	-2(2)	-1(2)	0(2)	-6	-4	-4	-5
c_{46}	2(2)	4(1)	5(1)	11	13	14	13
c_{55}	50(2)	51(2)	48(3)	29	41	44	48
c_{56}	0(2)	-2(1)	-2(2)	-5	-5	-5	-5
c_{66}	53(4)	53(1)	53(1)	49	49	49	48
B	88(1)	77(2)	75(1)	77(1)	72(2)	72(2)	71(2)

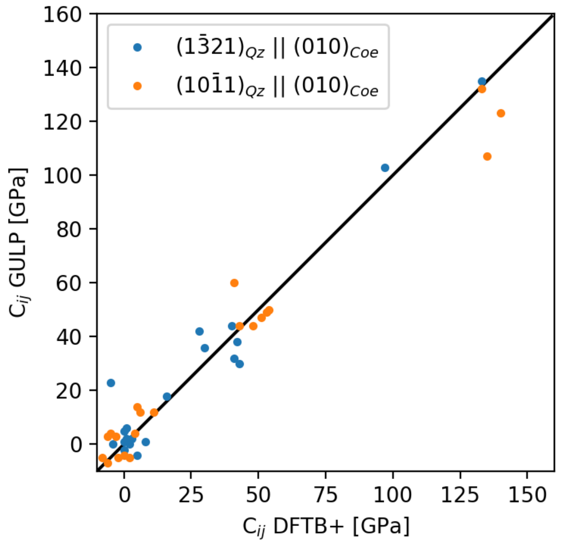

Fig. S1. Comparison of calculated elastic stiffness coefficients for interface structures which were formed by supercells built from three quartz and three coesite transformation cells.
